# A Pragmatic Approach to the Management of Severe Awake Bruxism in an Adolescent with Cerebral Palsy and Global Developmental Delay

**DOI:** 10.1155/2022/5288515

**Published:** 2022-01-13

**Authors:** N. Ismail, S. H. Hamzah, I. Wan Mokhtar

**Affiliations:** ^1^Centre for Paediatric Dentistry & Orthodontics Studies, Faculty of Dentistry, Universiti Teknologi MARA, Sungai Buloh Campus, Jalan Hospital, 47000 Sungai Buloh, Selangor, Malaysia; ^2^Ministry of Health, Malaysia; ^3^Centre for Comprehensive Care Studies, Faculty of Dentistry, Universiti Teknologi MARA. Sungai Buloh Campus, Jalan Hospital, 47000 Sungai Buloh, Selangor, Malaysia

## Abstract

Cerebral palsy is a neurological and motor condition characterised by muscle balance and posture impairments. Bruxism and malocclusion were frequently observed in patients with cerebral palsy, in contrast to other oral anomalies. The report outlines how severe awake bruxism is managed in a 16-year-old Korean boy who has nonverbal spastic cerebral palsy and global developmental delay. The treatment protocol involved the fabrication of soft occlusal splints of three and four millimetres in thickness, followed by the placement of stainless-steel crowns on all first permanent molars whilst video recording and a bruxism diary was kept. Fixed restorations demonstrate increased endurance in withstanding bruxism force in persons who are dependent on their caretaker.

## 1. Introduction

Cerebral palsy (CP) is a neurological disorder characterised by disturbances in muscular control that occur during pregnancy, perinatal life, or postnatal life [1]. The Surveillance of Cerebral Palsy in Europe (SCPE) has agreed to define CP as a group of permanent, but not irreversible, disorders of movement and posture, as well as motor function, caused by a nonprogressive interference, lesion, or abnormality of the developing/immature brain [2]. The prevalence of this clinical condition has been estimated to be two children per 1,000 births with a preference for males and African American populations [3]. Cerebral palsy is classified based on the nature of the motor symptoms (spastic, dyskinetic, or ataxic) and the degree of impairment (hemiplegia, diplegia, or tetraplegia).

When the masticatory muscles are harmed, oral health may be compromised as a result of altered orofacial structure, the development of parafunctional behaviours, nutritional problems, and difficulties with oral hygiene care [4]. Individuals with CP have a greater proclivity for delayed permanent molar eruption, Angle's Class II malocclusion [5, 6], dental trauma [7], and bruxism [8].

Bruxism is an affliction of a repetitive jaw-muscle activity characterised by tooth clenching or grinding and mandible bracing or thrusting. Bruxism manifests itself in two distinct circadian patterns: during sleep (referred to as sleep bruxism) or during wakefulness (referred to as awake bruxism) [9]. This repetitive grinding and gnashing can result in masseter muscle hypertrophy, headaches, temporomandibular disorder (TMD), and tooth abrasion [10]. Instrumental and noninstrumental approaches can be used to assess bruxism [11]. Instrumental approaches include the use of polysomnography (PSG) with audiovisual (AV) recording or a portable electromyograph (EMG) device to monitor the patient's masticatory muscle activity [12]. Noninstrumental approaches, on the other hand, were based on self-report (clinical history or questionnaires) and clinical examination, such as multifacet tooth wear [12]. Prevalence of bruxism in children with cerebral palsy ranges from 25.0% to 69.4% [8].

Additionally, dental attrition/erosion, a possible consequence of spasticity/bruxism, is significantly greater in subjects with CP than in age-matched controls [13]. Reduced and altered saliva production, as well as frequent gastroesophageal reflux, also contribute to dental attrition/erosion [14, 15]. The following case study and discussion detail the treatment given to a teenager with CP and global developmental delay (GDD) who suffered from severe awake bruxism.

## 2. Case Report

A sixteen-year-old Korean boy presented to the Special Care Dentistry Clinic (for children), with spastic cerebral palsy (type quadriplegia) and global developmental delay diagnosed at the age of three months. He arrived with his mother in a wheelchair and was nonverbal. The mother concerned of his continuous grinding and clenching behaviours especially when he is stressful, which causing attrition and wear tooth facets generally on his dentition and generalised staining on the permanent tooth. The anamnesis reveals that the pregnancy and delivery were both normal, and he was born at full term. There was no family history of cerebral palsy or other hereditary disorders.

He had previously experienced multiple convulsive crises triggered by a high fever and flashing lights during his five years of life and was previously prescribed with an anticonvulsant medication (Carbamazepine) only during that period. He is closely monitored by a multidisciplinary team comprised of a paediatrician, physiotherapist, psychologist, and neurologist. Physical examination revealed a weight of 47 kilogrammes and a height of 160 centimetres. He had a combination of movement impairments caused by abnormal reflexes, limb and trunk rigidity, abnormal posture, involuntary movements, and unsteady walking, which necessitated his reliance on a wheelchair for mobility. On a dietary note, he ate mostly semiblended or pureed foods.

**Figure 1:**
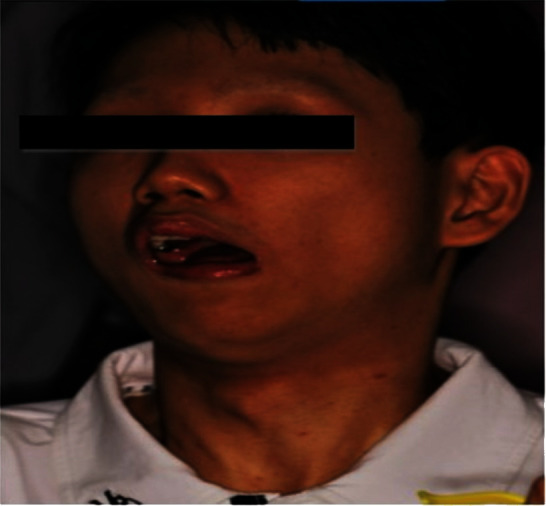
Extraoral photo of the patient.

**Figure 2:**
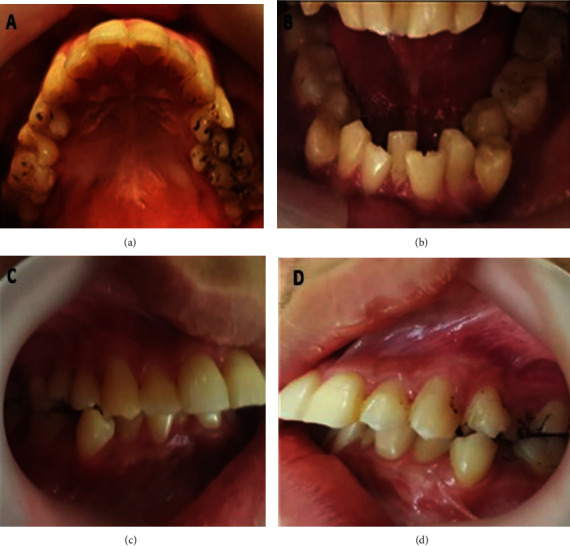
(a) Intraoral photograph of upper occlusal view showing generalised blackish staining on posterior teeth. (b) Intraoral photograph of lower occlusal view showing severe crowding complicated with a fusion of tooth 31 and tooth 32. (c) Intraoral photograph (right buccal view) showing buccally placed tooth 44, severely attritted incisal tip of 13. (d) Intraoral photograph (left buccal view) showing scissor bite between upper and lower first premolar and severe incisor overjet.

Extraoral palpation reveals hypertrophy of the masseter muscle on a class II skeletal pattern. Facial and hypoglossal nerve dysfunction were apparent, which exacerbates salivary drooling symptoms and poor in swallowing (Figure 1). The intraoral examination reveals a permanent dentition stage with optimal oral hygiene, visible generalised chromogenic bacteria with a blackish stain on the posterior tooth (Figure 2(a)). The blunt cusps on the first permanent molar and canine were evident. An incidental discovery of fusion tooth anomalies is observed on lower left incisors, teeth 31 and 32 (Figure 2(b)). Additionally, he had a Class II div I malocclusion, which included a deep overbite (lower incisors impinging on the anterior palate), bilateral posterior crossbite associated with a buccally displaced first lower premolar, and severe crowding on the lower anterior segment (Figures 2(c) and 2(d)). Radiographic examination was not possible due to the patient's unwillingness to cooperate during the acquisition.

## 3. Clinical Management

**Figure 3:**
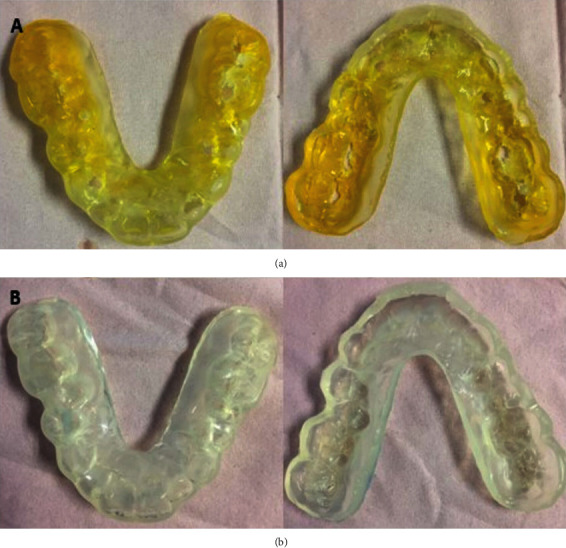
(a) Upper and lower soft occlusal splint wore during daytime. (b) Upper and lower soft occlusal splint wore at sleep-time.

The treatment management started with a 3 mm thick oral protective appliance (occlusal soft splint) fabrication. Impressions with alginate material were taken using a passive immobilisation technique. The splint issued within 2 weeks, with reassurance on the importance of proper oral hygiene. Unfortunately, patient returned within a month with a fractured split. Henceforth, a millimetre thicker (4 mm) splint is fabricated. Two splints were provided for daytime (Figure 3(a)) and another for sleep-time (Figure 3(b)).

The patient was followed up on a regular basis to remove and clean the appliances, apply topical fluoride, and be reminded to practise good oral hygiene. After a year of observation, the bruxism behaviour decreased, but the intensity is somewhat unchanged. It is noticed that the bruxism is more intense during daytime comparing to the nighttime as the daytime splint exhibits more wear off surfaces. As a result, a decision was made to increase the vertical height of the first permanent molars and thus reduce premature contacts. This is to achieve by placing stainless-steel crown (SSC) on the permanent molars.

**Figure 4:**
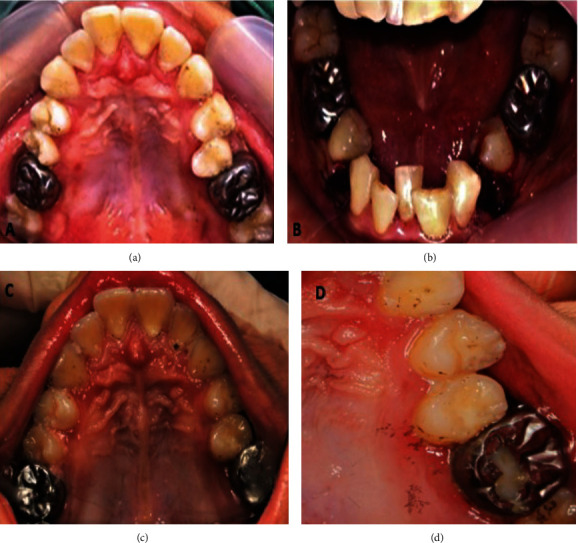
(a) Intraoperative upper occlusal views of cemented of stainless-steel crown on tooth 16 and tooth 26. (b) Intraoperative lower occlusal view showing mandibular first premolars were extracted and indentation on incisal edge of fusion teeth 31 and 32 was restored with composite resin. (c) Upper occlusal view after 7-month follow-up showing intact stainless-steel crowns (SSCs) on tooth 16 and tooth 26 with evidence of attritted crown on 26. (d) Intraoral pictures focusing on tooth 26 revealed that palatal cusp of tooth 26 is severely attritted and exposing the cement of the crown after 7-month follow-up.

Due to his unfavourable behaviour, the procedures were scheduled under general anaesthesia (GA). To engage the optimal occlusal force, a thorough occlusal analysis was performed prior in the dental office and repeated under the influence of GA. The distal cusps of the second permanent molars are submerged; thus, the crown is placed only on the first permanent molars. The crowded lower anterior segment, which contributed to the premature contact, was relieved by extracting both lower right and left first permanent premolars that were buccally displaced. Additionally, the tooth appears to complicate oral hygiene measures, justifying its extraction. The incisal third of the fusion tooth's indentation was restored using composite resin as a preventive resin restoration (Figures 4(a) and 4(b)).

## 4. Review and Maintenance

Postoperatively, the patient was on close monitoring regime; First visit after 1 month, 2nd to 4th visit in every 3 months, followed by 5th visit after 6 month until the condition deemed nonaggressively progressive. Within the first seven-month review visit, the mother reported that his son continued to have bruxism, but only when he was stressed or bored. Intraoral examination revealed that only the palatal cusp of SSC placed on the upper left first permanent molar tooth (26) attritted, exposing the underlying GIC cement (Figures 4(c) and 4(d)). The progression of attrition was monitored until there is need for replacement. Both parents and clinician decided on starting on a bruxism diary to engage with the habit intensity and timing. The mother agreed and advised on video recording with written log capturing the timing, frequency, and severity of the habit. The records would aid in identifying the specific stressors that contribute to the parafunctional habits, allowing for a better understanding and intervention to the habit.

## 5. Discussion

Various modalities of treatment for the management of bruxism have been investigated such as occlusal intervention (either by occlusal appliance or “Nociceptive Trigeminal Inhibition (NTI) Clenching Suppression System”—a small anterior splint). Biofeedback therapy is based on the principle that bruxers can “unlearn” their behaviour when a stimulus makes them aware of their adverse jaw muscle activities (“aversive conditioning”) and pharmacological approach like antidepressant drugs (benzodiazepine or L-dopa) or drugs that have paralytic effect on the muscle through an inhibition of acetylcholine release at the neuromuscular junction (botulinum toxin) which decreases bruxism activity [16].

Bruxism was diagnosed in this patient using noninstrumental methods, where self-reported signs and symptoms by mother as well as clinical evidence of generalised attrition. Despite the low concordance with instrumental assessment, particularly in assessing sleep bruxism [17], self-reported assessment may be entirely valid for a particular application and remains the primary tool in research and clinical practise [12]. After the treatment with soft splint, the study learned that his awake bruxism is more intense comparing to the sleep bruxism. This is concluded based on the attrition evidence difference between both splints as suggested by Koyano et al. [18].

A systematic review by Canales et al. [19] showed that most studies using botulinum toxin injection focused on sleep bruxism, whilst awake bruxism has been addressed only with single case reports. Botulinum toxin injection may influence only the last phase of a sleep bruxism episode, by reducing the intensity of the contraction, whilst it cannot have any effects on its genesis [19]. It should also be noted that, despite awake bruxism is mainly related to anxiety or daily stress [20], some case reports showed that botulinum toxin injection in patients with severe awake bruxism episodes may be an alternative option to wearing oral appliances [21]. Despite botulinum toxin has been increasingly diffused in dentistry over recent years particularly for pain management in patient with bruxism, nonetheless, there is no consensus about its effects in this disorder as lack of clinical protocols, standardization of dosage, and different dilution of preparations between the various commercial brands may contribute to explain the incomplete knowledge on the topic [22].

Dental treatment of bruxism with an interocclusal appliance (splint) is based on Glaros and Melamed's local or mechanical theories [23]. This theory postulated that bruxism may be caused by tooth failing to achieve optimal intercuspal position or retruded cuspal position. Deviation from the ideal structure impairs the nervous feedback system and proprioceptive receptors in the mandible, lowering the irritability threshold, and bruxism occurs as a teleological response to restore ideal occlusion. Interocclusal appliances have been shown to reduce bruxing activity by decreasing bruxing frequency but not duration or amplitude [24].

In the initial treatment, the patient is prescribed with soft split (silicone type) to cover both maxillary and mandibular teeth, providing a cushion effect during intercuspation and preventing them from contacting one another, thereby preventing damage caused by the grinding habit and reducing masticatory activity [25]. We decided to use silicon-type material which is based on the study by Seifeldin and Elhayes that shows the use of soft occlusal splint exhibits superior and earlier improvement of some temporomandibular joint disorder (TMD) [26]. It was demonstrated that 3 mm splints can be used effectively in the treatment of bruxism as it is superior in comfort and has remarkable result for TMD compared to 5 mm and 6 mm splints [27, 28]. However, over the time, the soft splint became worn out and the second devices were constructed from the same material with increased occlusal height of 4 millimetres. Hegab et al. found that 4 mm splint therapy has as nearly as effectiveness like 3 mm splint therapy [29].

The consideration for a reversible and conservative treatment is crucial to avoid unnecessary treatment. However, the therapeutic effect appears to be limited to the duration of the device's ability to maintain the bruxism habit's frequency and usually recommended for short-term use because degradation can occur rapidly [30]. Due to the guarded changes in the grinding frequency, the treatment switched to a stainless-steel crown (SSC). SSC is shown to be less technique sensitive and can reduce chairside time which is an important consideration in managing patient with disability [31].

According to a previous study, the increased prevalence of Angle's Class II is due to an imbalance between the perioral and intraoral muscles, as evidenced by unusual tongue movement or positioning [6]. Bilateral extraction of buccally located lower first premolars was performed in this patient to alleviate lower anterior crowding and improve oral hygiene.

Numerous studies have attempted to establish a link between stress and bruxism. Children who brux experienced a greater level of stress than children who did not brux and bruxers were significantly more likely to experience increased levels of anxiety [32]. Vanderas et al. [33] discovered that children with significantly elevated stress levels had significantly higher catecholamine levels in their urine and a higher prevalence of bruxism [33]. The children's bruxism diary suggested to identify risk factors for bruxism. Aspects of behaviour such as anxiety management and occupying the patient's time with something interesting may aid in the treatment of this condition [34].

## 6. Conclusion

In conclusion, the occlusal splint is a reversible intervention that acts as a provisional treatment for tooth grinding and tooth wear control. We foresee that by placing stainless-steel crown on first permanent molar once it is fully erupted may be a promising prophylactic measure in patient with cerebral palsy (CP) and global developmental delay (GDD).

## Data Availability

No data were used to support this study.
